# Characterization of Novel ACE-Inhibitory Peptides from *Nemopilema nomurai* Jellyfish Venom Hydrolysate: In Vitro and In Silico Approaches

**DOI:** 10.3390/md23070267

**Published:** 2025-06-26

**Authors:** Ramachandran Loganathan Mohan Prakash, Deva Asirvatham Ravi, Du Hyeon Hwang, Changkeun Kang, Euikyung Kim

**Affiliations:** 1 College of Veterinary Medicine, Gyeongsang National University, Jinju 52828, Republic of Korea; mohan_22@gnu.ac.kr (R.L.M.P.); devabiochem@gnu.ac.kr (D.A.R.); pooh9922@hanmail.net (D.H.H.); ckkang@gnu.ac.kr (C.K.); 2 Institute of Animal Medicine, Gyeongsang National University, Jinju 52828, Republic of Korea

**Keywords:** angiotensin-converting enzyme inhibitors, peptide characterization, Lineweaver–Burk plot, molecular docking and dynamics, network pharmacology

## Abstract

The venom of *Nemopilema nomurai* jellyfish represents a promising source of bioactive compounds with potential pharmacological applications. In our previous work, we identified two novel angiotensin-converting enzyme (ACE)-inhibitory peptides—IVGRPLANG (896.48 Da) and IGDEPRHQYL (1227.65 Da)—isolated from *N. nomurai* venom hydrolysates via papain digestion. In this study, we conducted a detailed biochemical and computational characterization of these peptides. The IC_50_ values were determined to be 23.81 µM for IVGRPLANG and 5.68 µM for IGDEPRHQYL. Kinetic analysis using Lineweaver–Burk plots revealed that both peptides act as competitive ACE inhibitors, with calculated inhibition constants (K_i_) of 51.38 µM and 5.45 µM, respectively. To assess the structural stability of the ACE–peptide complexes, molecular dynamics simulations were performed. Root mean square deviation (RMSD) and root mean square fluctuation (RMSF) analyses provided insights into complex stability, while interaction fraction analysis elucidated key bond types and residue–ligand contacts involved in binding. Furthermore, a network pharmacology approach was employed to predict therapeutic targets within the renin–angiotensin–aldosterone system (RAAS). Eleven target proteins were identified: IVGRPLANG was associated with REN, ACE, CTSB, CTSS, and AGTR2; IGDEPRHQYL was linked to REN, AGT, AGTR1, AGTR2, KNG1, and BDKR2. Molecular docking analyses using HADDOCK software (version 2.4) were conducted for all targets to evaluate binding affinities, providing further insight into the peptides’ therapeutic potential.

## 1. Introduction

Hypertension, often referred to as the “silent killer”, is characterized by an elevated systolic blood pressure (SBP) ≥140 mmHg or diastolic blood pressure (DBP) ≥90 mmHg [1]. It is the most significant risk factor for cardiovascular disease (CVD) globally and is considered a serious chronic condition due to the persistent elevation of blood pressure levels [2,3,4,5]. Furthermore, hypertension is responsible for approximately 54% of stroke-related deaths and 45% of cardiovascular-related deaths in East Asian countries [6]. To address this global health challenge, the World Health Assembly established a target in 2013 to reduce the prevalence of elevated blood pressure by 25% by the year 2025, as part of its broader non-communicable disease (NCD) reduction goals. According to self-reported data from a large-scale survey involving 533,306 adults, eliminating hypertension in women could potentially reduce overall population mortality by 7.3%, compared to 0.1% for hyperlipidemia, 4.1% for diabetes, 4.4% for smoking, and 1.7% for obesity. In men, the corresponding reductions in mortality would be 3.8% for hypertension, 2.0% for hyperlipidemia, 1.7% for diabetes, 5.1% for smoking, and 2.6% for obesity [7].

Among the various physiological pathways, the renin–angiotensin–aldosterone system (RAAS) plays a critical role in maintaining hemodynamic stability by regulating blood pressure, fluid volume, and the balance of sodium and potassium. Consequently, any imbalance in the molecular components of RAAS can contribute to the pathogenesis of hypertension [8]. Within this system, angiotensin-converting enzymes (ACEs) are a key regulator of blood pressure. ACEs catalyze the conversion of angiotensin I to angiotensin II, a potent vasoconstrictor, thereby contributing to vascular resistance and elevated blood pressure [9,10,11]. ACE inhibitors are widely prescribed for the treatment of hypertension and various cardiovascular conditions and are considered among the most effective classes of antihypertensive agents [12,13].

Since the U.S. Food and Drug Administration (FDA) approved captopril in 1981—the first ACE inhibitor derived from a peptide analog found in snake venom—other inhibitors such as enalapril and lisinopril have been developed and extensively used in clinical settings. However, these synthetic drugs are often associated with adverse side effects, prompting a growing interest in identifying alternative, naturally derived ACE-inhibitory compounds [14]. In recent years, a number of studies have focused on the discovery of ACE inhibitors from natural sources. Notably, the enzymatic hydrolysis of marine organisms has yielded promising bioactive peptides with ACE-inhibitory activity. These sources include *Acaudina molpadioidea* (sea cucumber), mussels, salmon skin, squid skin, shrimp, and jellyfish [15,16,17,18,19,20]. These findings underscore the potential of marine-derived peptides as therapeutic agents for managing hypertension and associated cardiovascular risks.

Jellyfish possess numerous specialized cells known as cnidocytes, primarily located in their tentacles. These cells discharge venom through a specialized organelle called the nematocyst. Beyond their well-known toxic effects, jellyfish venoms also contain a variety of pharmacologically active proteins and peptides with potential therapeutic applications [21,22,23,24]. Several studies have identified angiotensin-converting enzyme (ACE)-inhibitory peptides from jellyfish venom, including those from *Chiropsalmus quadrigatus* [25] and *Nemopilema nomurai* [26]. In our previous study [26], we isolated two novel ACE-inhibitory peptides from the papain hydrolysate of *N. nomurai* jellyfish venom. Sequential chromatographic techniques were employed to purify the peptides, and their ACE-inhibitory activities were evaluated in vitro. The peptide sequences were determined via LC-MS/MS analysis and identified as IVGRPLANG (896.48 Da) and IGDEPRHQYL (1227.65 Da). Furthermore, molecular docking analysis was conducted to assess the binding affinities of these peptides to the ACE, supporting their potential as natural ACE inhibitors [26].

The objective of this study is to characterize comprehensively the two ACE-inhibitory peptides using both in vitro and in silico approaches. Specifically, we evaluated their inhibitory potency (IC_50_), mode of inhibition, and stability against gastrointestinal digestive enzymes. In addition, molecular dynamics (MD) simulations were conducted to assess the structural stability of the ACE–peptide complexes (ACE–IVGRPLANG and ACE–IGDEPRHQYL). A network pharmacology approach was employed to identify potential hypertension-related target proteins, followed by molecular docking to evaluate peptide–target interactions. Overall, this study provides deeper insight into the pharmacological potential of jellyfish venom-derived peptides and supports their possible application in the development of novel antihypertensive therapeutics.

## 2. Results

### 2.1. ACE-Inhibitory Activity and Resistance to Simulated GI Digestion of IVGRPLANG and IGDEPRHQYL Peptides

**Figure 1:**
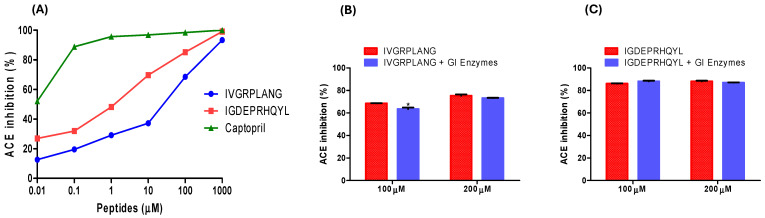
(**A**) ACE-inhibitory activity of IVGRPLANG and IGDEPRHQYL peptides, with captopril as a positive control. Resistance to simulated GI digestion assay of peptides (**B**) IVGRPLANG and (**C**) IGDEPRHQYL using gastrointestinal (GI) enzymes. The results are expressed as mean ± standard (SD), with significance denoted by * *p *< 0.05 (µM: concentration).

The ACE-inhibitory activity of the peptides was evaluated in vitro using the Dojindo assay kit, following the same procedure as described in our previous study [27]. Both peptides exhibited dose-dependent ACE-inhibitory effects (Figure 1A). The half-maximal inhibitory concentrations (IC_50_) were determined to be 23.81 µM for IVGRPLANG and 5.68 µM for IGDEPRHQYL. Captopril was used as a positive control to validate the assay system. To assess the stability of the peptides under simulated gastrointestinal (GI) conditions, a two-step enzymatic hydrolysis was conducted. The ACE-inhibitory activity of the peptides was measured both before and after digestion (Figure 1B,C). There were no statistically significant differences in ACE-inhibitory activity before and after GI digestion, suggesting that both peptides exhibit strong resistance to enzymatic degradation under gastrointestinal conditions.

### 2.2. Inhibition Pattern of IVGRPLANG and IGDEPRHQYL Peptides

**Figure 2:**
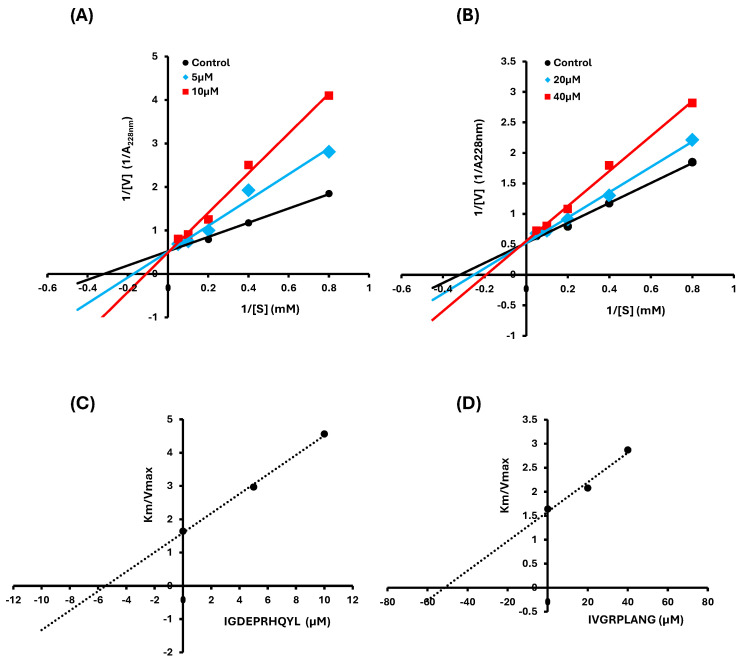
Lineweaver–Burk plot and secondary plot for IVGRPLANG (**A**,**C**) and IGDEPRHQYL (**B**,**D**), respectively (µM and mM: concentration).

Lineweaver–Burk plots were used to determine the mode of inhibition exerted by the peptides. Both IVGRPLANG and IGDEPRHQYL exhibited a competitive inhibition pattern with respect to the substrate, indicating that they bind to the active site of ACE. The Michaelis–Menten constants (Km) and maximum reaction velocities (Vmax) were derived from the plots (Figure 2A,B). These results support the conclusion that both peptides act as competitive inhibitors of ACE. Furthermore, secondary plots were constructed to calculate the inhibition constant (Ki). The Ki values were determined to be 51.389 µM for IVGRPLANG and 5.457 µM for IGDEPRHQYL (Table 1), further confirming their respective inhibitory potencies.

### 2.3. In Silico Screening of IVGRPLANG and IGDEPRHQYL Peptide with Angiotensin-Converting Enzyme

**Figure 3:**
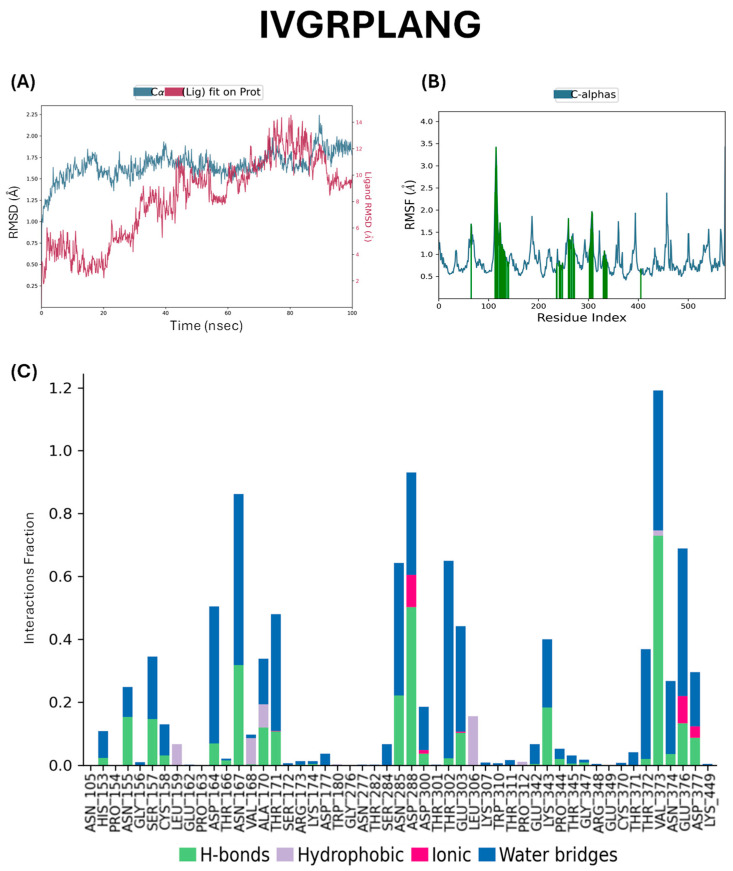
Molecular dynamics of IVGRPLANG peptide with angiotensin-converting enzyme. (**A**) RMSD, (**B**) RMSF, (**C**) interaction fractions. Green bars below the RMSF plot indicate residues interacting with the ligand, particularly those located in or near the active binding pocket (Å: Angstrom, unit for reporting molecular dynamics distances).

**Figure 4:**
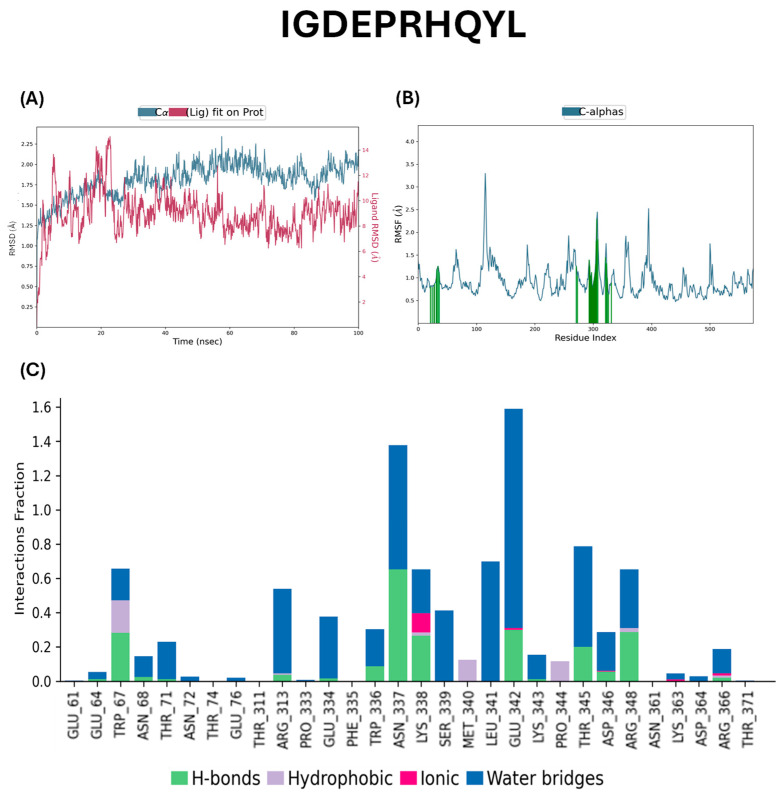
Molecular dynamics of IGDEPRHQYL peptide with angiotensin-converting enzyme. (**A**) RMSD, (**B**) RMSF, (**C**) interaction fractions. Green bars below the RMSF plot indicate residues interacting with the ligand, particularly those located in or near the active binding pocket (Å: Angstrom, unit for reporting molecular dynamics distances).

Molecular dynamics (MD) simulations were performed to assess the binding stability of the ACE protein in complex with the peptides IVGRPLANG and IGDEPRHQYL (Figure 3 and Figure 4). Root mean square deviation (RMSD) analysis was conducted to evaluate the conformational stability of the protein–ligand complexes over a 100 ns simulation period. As shown in Figure 3A and Figure 4A, the RMSD evolution of the ACE backbone (left *Y*-axis) indicates that both systems reached equilibrium, with fluctuations stabilizing toward the end of the simulation. In the ACE–IVGRPLANG complex, protein RMSD values ranged from a minimum of 0.892 Å to a maximum of 2.242 Å, suggesting minimal deviation and high structural stability. Similarly, in the ACE–IGDEPRHQYL complex, protein RMSD values ranged from 1.083 Å to 2.178 Å, indicating comparable backbone stability. The ligand RMSD (right *Y*-axis) reflects how stable the peptide remains within the binding pocket. “Lig fit Prot” represents the RMSD of the heavy atoms ligand after the complex is aligned to the protein backbone. When ligand RMSD values remain within the same range as the protein’s RMSD, it implies that the ligand remains stably bound throughout the simulation. In Figure 3A, the IVGRPLANG–protein complex exhibited continuous fluctuations throughout the 100 ns simulation, indicating ongoing conformational adjustments and the absence of complete structural stabilization. For IGDEPRHQYL, initial fluctuations were slightly lower and stabilized after approximately 20 ns. Notably, the ligand–protein complex involving IGDEPRHQYL exhibited a narrower fluctuation range (~8–10 Å) compared to the IVGRPLANG complex, suggesting relatively improved binding stability over the course of the simulation.

Root mean square fluctuation (RMSF) analysis was performed to evaluate the local flexibility of individual amino acid residues within the ACE protein during complex formation with the peptides (Figure 3B and Figure 4B). RMSF provides insights into the dynamic behavior of residues throughout the simulation, with peaks in the plot representing regions of higher atomic fluctuation. Additionally, the RMSF analysis (Figure 3B and Figure 4B) revealed increased flexibility in residues located near the binding site, particularly for IVGRPLANG (His113, Asn115, Ser255, Met259, Glu263, Glu302, and Lys303 in Figure 3B) and for IGDEPRHQYL (Thr34, Glu36, Thr305, Asp306, Lys323, and Asp324 in Figure 4B). These residues correspond to regions involved in peptide interactions, suggesting that local flexibility may contribute to the dynamic nature of peptide binding and influence binding affinity. As expected, the N- and C-terminal regions of the protein exhibited higher flexibility compared to the more rigid secondary structural elements such as α-helices and β-strands. These structured regions typically demonstrate lower RMSF values, whereas loop regions tend to fluctuate more due to their unstructured nature. For the IVGRPLANG–ACE complex (Figure 3B), RMSF values ranged from 0.553 Å to 3.424 Å, while for the IGDEPRHQYL–ACE complex (Figure 4B), the values ranged from 0.585 Å to 3.335 Å. These results indicate that both peptide–protein complexes maintained overall structural stability, with only localized flexibility observed in terminal and loop regions.

Protein–ligand interaction profiles for the ACE–peptide complexes were analyzed throughout the molecular dynamic simulation, with interacting residues represented by green vertical bars in Figure 3C and Figure 4C. These interactions were categorized into four main types: hydrogen bonds, hydrophobic interactions, ionic interactions, and water bridges. Interaction frequency is displayed in normalized stacked bar charts across the simulation trajectory. For instance, a value of 0.7 indicates that a specific interaction was maintained for 70% of the simulation time. Values exceeding 1.0 are possible when a single residue forms multiple interactions of the same type with the ligand. Interaction definitions used in this analysis were as follows: Hydrogen Bonds: A donor–acceptor distance ≤ 2.5 Å, donor angle ≥ 120°, and acceptor angle ≥ 90°. Hydrophobic Interactions: π-cation, aromatic and charged groups within 4.5 Å. π–π interactions: Face-to-face or face-to-edge aromatic group stacking. Other hydrophobic interactions: Aliphatic/aromatic carbon interactions within 3.6 Å. Ionic Interactions: Oppositely charged atoms within 3.7 Å (not involved in hydrogen bonds), classified by involvement of protein backbone or side chain. Metal Coordination: A metal ion within 3.4 Å of both protein and ligand heavy atoms (excluding carbon). Water Bridges: Protein–water or water–ligand hydrogen bonds with donor–acceptor distance ≤ 2.8 Å, donor angle ≥ 110°, and acceptor angle ≥ 90°. The key interacting residues were selected that are greater than 0.6 interaction fraction for both peptides between ACE proteins are given as follows: IVGRPLANG: ASN167, ASN285, ASP288, THR302, VAL373, and GLU376 (Figure 3C); IGDEPRHQYL: TRP67, ASN337, LYS338, LEU341, GLU342, THR345, and ARG348 (Figure 4C). These results indicate strong and persistent interactions between the peptides and ACE, supporting their stable binding behavior observed in RMSD and RMSF analyses. The molecular dynamics simulation was performed only once. We acknowledge this limitation and plan to perform repeated simulations in future studies to further validate our findings. Nevertheless, we believe that the current results offer meaningful insights into the system’s behavior.

### 2.4. Exploring Potential Hypertension-Related Targets for IVGRPLANG and IGDEPRHQYL Peptides and Performing GO and KEGG Analyses

**Figure 5:**
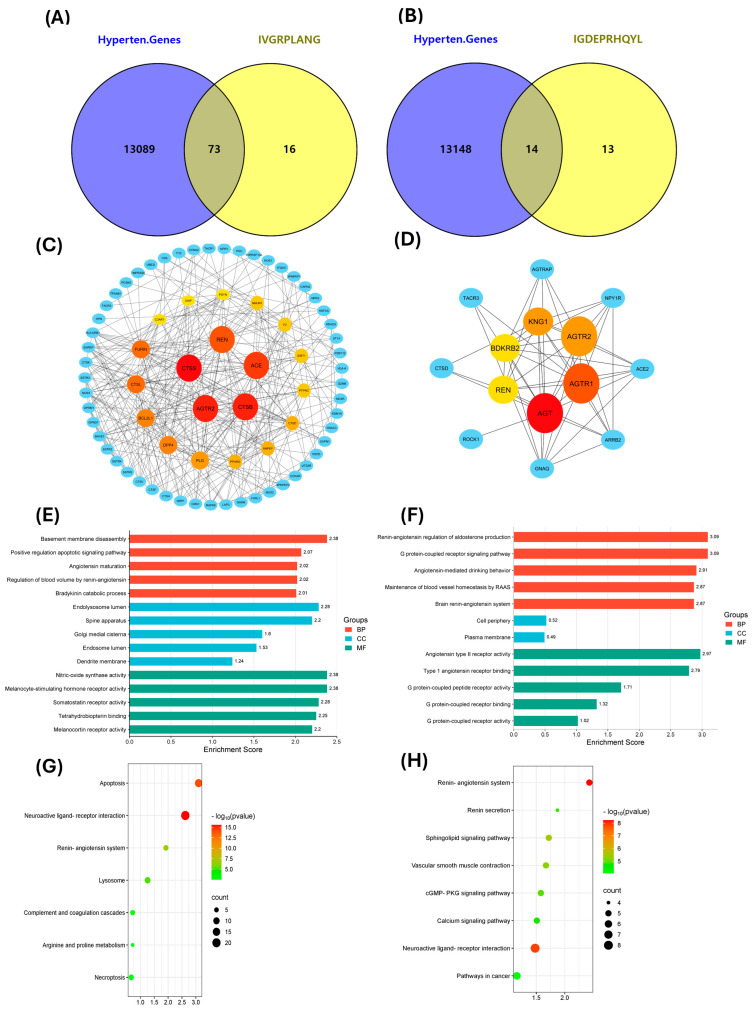
Network pharmacology analysis for IVGRPLANG and IGDEPRHQYL. (**A**,**B**) Common hypertension targets. (**C**,**D**) Protein–protein interactions using hypertension targets. (**E**,**F**) KEGG pathway enrichment analysis. (**G**,**H**) GO analysis of hypertension targets.

The pathogenesis of hypertension is multifactorial, and most commercially available antihypertensive drugs are designed to act on a single molecular target, often resulting in undesirable side effects. To investigate whether the venom-derived peptides IVGRPLANG and IGDEPRHQYL exhibit multitarget potential, a hypertension-related gene library was constructed using a network pharmacology approach. Predicted targets of the ACE-inhibitory peptides were identified and compared to known hypertension-associated genes. This comparison revealed 73 common targets for IVGRPLANG (Figure 5A) and 14 for IGDEPRHQYL (Figure 5B). The resulting gene sets were uploaded to the STRING database for protein–protein interaction (PPI) analysis. In the resulting PPI networks, larger node sizes correspond to higher degree values, indicating more highly connected and potentially central proteins in the network.

Among the 73 IVGRPLANG-associated targets, Renin (REN), Angiotensin-Converting Enzyme (ACE), Cathepsin B (CTSB), Cathepsin S (CTSS), and Angiotensin II Receptor Type 2 (AGTR2) were identified as highly ranked nodes (Figure 5C). Similarly, in the IGDEPRHQYL target set, Angiotensinogen (AGT), Angiotensin II Receptor Type 1 (AGTR1), AGTR2, REN, Kininogen I (KNG1), and Bradykinin Receptor B2 (BDKRB2) emerged as prominent targets (Figure 5D). Gene Ontology (GO) enrichment analysis (Figure 5E,F) and Kyoto Encyclopedia of Genes and Genomes (KEGG) pathway analysis (Figure 5G,H) further demonstrated that the identified targets of both peptides are significantly involved in pathways related to blood pressure regulation. These include the renin–angiotensin system, renin secretion pathway, blood vessel homeostasis, angiotensin maturation, nitric oxide biosynthesis, and angiotensin receptor-binding activity, highlighting the peptides’ potential as multitarget antihypertensive agents.

### 2.5. Docking Studies of Potential Hypertensive Target Proteins with IGDEPRHQYL and IVGRPLANG Peptide

**Figure 6:**
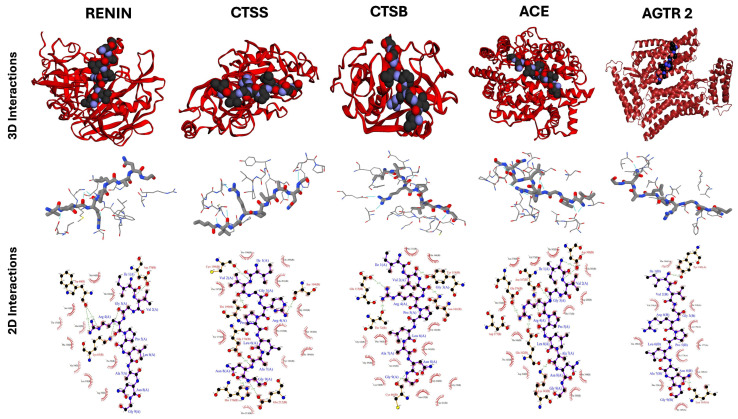
Molecular docking interactions of IVGRPLANG with hypertension target proteins.

**Figure 7:**
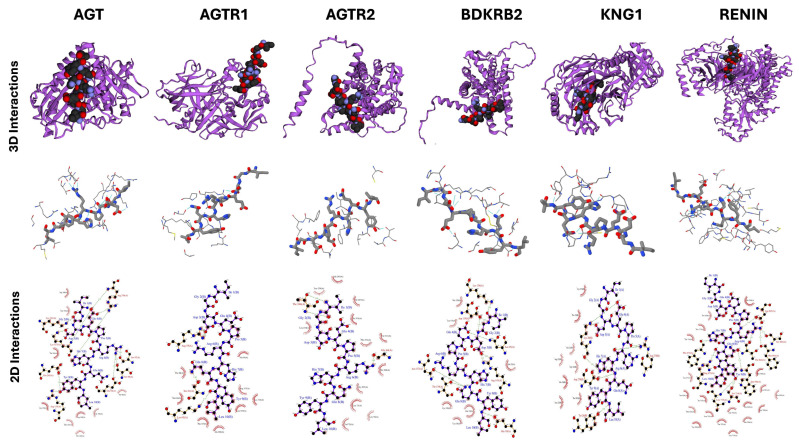
Molecular docking interactions of IGDEPRHQYL with hypertension target proteins.

The binding interactions of the peptides IVGRPLANG and IGDEPRHQYL with key hypertension-related targets were further examined through molecular docking analysis. These peptides were docked with high-degree nodes identified in the protein–protein interaction (PPI) network, including REN, ACE, CTSB, CTSS, and AGTR2 for IVGRPLANG, and AGT, AGTR1, AGTR2, REN, KNG1, and BDKRB2 for IGDEPRHQYL. Target selection was based on their network centrality, reflecting potential regulatory importance in hypertensive signaling pathways. The docking results, summarized in Table 2 and visualized in Figure 6 and Figure 7, provide insights into the binding affinities and interaction stabilities of the peptide–protein complexes. Binding energy values, represented by HADDOCK scores, serve as indicators of spontaneous interaction, where more negative values suggest stronger and more stable binding (i.e., binding energy < 0 kcal/mol). For IVGRPLANG, the HADDOCK binding scores were REN: −65.4, ACE: −54.5, CTSB: −27.8, CTSS: −17.2, and AGTR2: −29.5. For IGDEPRHQYL, the scores were AGT: −72.2, AGTR1: −60.3, AGTR2: −36.3, REN: −67.3, KNG1: −87.4, and BDKRB2: −115.3. In HADDOCK, more negative scores represent stronger and more favorable binding interactions, as the score is a weighted sum of van der Waals, electrostatic, desolvation, and restraint energy terms. In addition to the HADDOCK binding scores, relevant docking parameters—including top-hit cluster size, number of models per cluster, overall RMSD values, and Z-scores for each target complex—are summarized in Table 2. The additional data support the structural and functional evaluation of multiple hypertension-related target proteins and their interactions with the identified ACE-inhibitory peptides, IVGRPLANG and IGDEPRHQYL. Structural models for key targets, including BDKRB2 and RENIN, were generated using ColabFold (https://colab.research.google.com/github/sokrypton/ColabFold/blob/main/AlphaFold2.ipynb, accessed on 24 June 2025). The top-ranked models were selected based on pLDDT and pTM scores, indicating high structural reliability. Predicted binding pockets from PrankWeb aligned with known active site residues, suggesting that docking analyses were focused on catalytically relevant regions. The HADDOCK docking results—including favorable binding energies, detailed cluster characteristics, high buried surface areas, and low RMSD values—collectively support the formation of stable peptide–protein complexes and are given in Figures S1 and S2 and Tables S1–S4.

Among the docked complexes, IVGRPLANG showed the weakest binding affinity with Cathepsin B (CTSB), followed by AGTR2, as indicated by their relatively less negative HADDOCK scores. Similarly, IGDEPRHQYL exhibited the lowest binding affinity with AGTR2 among its target proteins. In contrast, the remaining targets demonstrated more favorable binding energies, supporting the predictive validity and consistency of the network pharmacology-based target selection. Figure 6 and Figure 7 present both three-dimensional (3D) and two-dimensional (2D) visualizations of the docking interactions between each peptide and its respective antihypertensive targets. These representations highlight hydrogen bonding as the predominant interaction type stabilizing the peptide–protein complexes.

## 3. Discussion

According to a 2020 report [27], several therapeutics have been successfully developed from animal-derived toxins, including captopril and enalapril (ACE inhibitors derived from the *Bothrops jararaca* pit viper), exenatide (an anti-diabetic drug originating from the Gila monster lizard), and tirofiban (an antiplatelet agent modeled after a component of the saw-scaled viper). These examples underscore the pharmacological potential of venom-derived bioactive compounds.

In this context, the present study provides a preliminary evaluation of the therapeutic applicability of peptides derived from *Nemopilema nomurai* jellyfish venom [26]. In our previous work, we isolated and identified two novel ACE-inhibitory peptides—IVGRPLANG and IGDEPRHQYL—from papain-hydrolyzed *N. nomurai* venom. However, their inhibitory potencies had not been quantitatively determined. To address this gap, both peptides were chemically synthesized with high purity (>95%) and subjected to comprehensive in vitro and in silico evaluations. The results revealed that both peptides exhibited notable ACE-inhibitory activity, with IGDEPRHQYL demonstrating greater potency (IC_50_ = 5.68 µM) compared to IVGRPLANG (IC_50_ = 23.81 µM). These findings are in line with previous reports of jellyfish-derived ACE-inhibitory peptides from species such as *Chiropsalmus quadrigatus* [25] and *Rhopilema esculentum* [20], further supporting the therapeutic potential of marine venom components. While additional studies using animal models are required to confirm the in vivo antihypertensive effects of these peptides [28], a major challenge in the development of peptide-based drugs is their susceptibility to degradation by gastrointestinal (GI) enzymes, which often compromises their efficacy following oral administration [29,30]. Therefore, assessing peptide stability during GI digestion is a critical step in early-stage drug development. Given the increasing ethical concerns regarding animal use and the high cost of preclinical trials, in vitro simulated gastrointestinal digestion (GID) models have gained broad acceptance as a predictive tool for evaluating in vivo peptide stability [31]. In this study, both IVGRPLANG and IGDEPRHQYL retained their ACE-inhibitory activity after GID treatment, indicating resistance to enzymatic degradation and suggesting structural stability under physiological digestive conditions. These results support their potential as orally administrable therapeutic agents, consistent with previous findings demonstrating that ACE-inhibitory peptides can maintain bioactivity following simulated digestion [32,33]. Although we strongly believe that the peptides are stable and biologically active based on our in vitro results, we plan to examine their structural stability after enzymatic digestion using LC-MS/MS or HPLC in the near future.

ACE-inhibitory peptides typically exert their effects through competitive, non-competitive, or mixed modes of enzyme inhibition [34]. In our study, Lineweaver–Burk plot analysis revealed that both peptides inhibit ACE via a competitive mechanism, as evidenced by an increase in Km values with increasing inhibitor concentration, while Vmax remained relatively constant. For IVGRPLANG, Km increased from 3.147 to 5.196 with minimal change in Vmax (1.919 to 1.810). IGDEPRHQYL exhibited a stronger competitive effect, with Km increasing from 3.147 to 9.198 and Vmax ranging from 1.919 to 2.015. These observations were further supported by the inhibition constants (Ki), where IGDEPRHQYL displayed a significantly lower Ki (5.457 µM) compared to IVGRPLANG (51.389 µM), confirming its stronger binding affinity and potency. Such competitive inhibition mechanisms are well documented among ACE inhibitors, including captopril and its analogs [35,36,37]. Taken together, these findings demonstrate that venom-derived peptides from *N. nomurai* exhibit promising pharmacological characteristics, positioning them as potential leads for future antihypertensive drug development.

Molecular dynamics (MD) simulation is a widely employed computational approach in modern drug discovery that enables the estimation of dynamic and thermodynamic properties of biomolecular systems under near-physiological conditions [38,39,40]. Accordingly, MD simulations were performed to validate the binding stability of the ACE–peptide complexes involving IVGRPLANG and IGDEPRHQYL. Root mean square deviation (RMSD) analysis revealed stable protein–ligand interactions for both complexes, with minimal fluctuations ranging between 1 and 2 Å, indicating strong binding affinity. Although initial fluctuations were observed, both complexes reached equilibrium over time, suggesting that the peptides remained stably bound within the ACE active site. It is generally accepted that a system can be considered equilibrated when the RMSD fluctuations are maintained within ±0.1 nm (1 Å) over time [41]. Root mean square fluctuation (RMSF) analysis revealed localized mobility primarily within loop regions, while α-helices and β-strands remained structurally stable. Notably, the IGDEPRHQYL–ACE complex exhibited slightly reduced RMSF values compared to the IVGRPLANG–ACE complex, suggesting greater conformational stability and stronger binding. The two peptides act as competitive inhibitors of ACE, as confirmed by 100 ns MD simulations. Both peptides formed stable complexes and interacted with residues located within or near the active site. Specifically, IVGRPLANG engaged Asn167, suggesting its direct occupation of the catalytic groove. IGDEPRHQYL interacted with Thr345 and Arg348, which are associated with substrate recognition. These interactions suggest that both peptides may interfere with substrate binding and exert their effects through competitive inhibition. In recent years, in silico techniques have emerged as powerful tools for characterizing protein–ligand interactions and gaining mechanistic insights into protein function and ligand specificity [42]. In the present study, molecular interaction analysis demonstrated that both peptides formed stable hydrogen bonds, hydrophobic contacts, ionic interactions, and water bridges with ACE. Among them, IGDEPRHQYL showed more extensive interactions with key active site residues, indicating a higher binding affinity and stronger interaction stability than IVGRPLANG. These computational findings confirm that both peptides form stable complexes with ACE, with IGDEPRHQYL displaying superior binding behavior. Furthermore, network pharmacology analysis revealed that IVGRPLANG and IGDEPRHQYL may interact with multiple hypertension-related targets, extending their therapeutic relevance beyond ACE inhibition. High-confidence targets identified in the PPI network included Renin (REN), Angiotensin-Converting Enzyme (ACE), Angiotensin II Receptors (AGTR1 and AGTR2), and Bradykinin Receptor B2 (BDKRB2)—all of which are well-established components of the renin–angiotensin–aldosterone system (RAAS) and play critical roles in blood pressure regulation [43]. Additional important targets included Kininogen I (KNG1) and the Cathepsins CTSB and CTSS. KNG1 is a key member of the kallikrein–kinin system, contributing to vasodilation and blood pressure modulation [44]. Cathepsins, particularly CTSB and CTSS, have been implicated in the pathogenesis of hypertension by promoting vascular smooth muscle proliferation, extracellular matrix degradation, cardiomyocyte hypertrophy, and the activation of the RAAS [45]. PPI analysis further confirmed the central role of these targets in the interaction network, and functional enrichment analysis showed their involvement in critical pathways such as the renin–angiotensin system, nitric oxide biosynthesis, and vascular homeostasis. Taken together, these in silico findings support the hypothesis that IVGRPLANG and IGDEPRHQYL possess multitarget potential as antihypertensive agents capable of modulating a broad range of hypertension-related pathways beyond ACE inhibition. However, molecular experiments must be conducted to experimentally validate these predicted hypertensive interaction targets in our future studies. Molecular docking analysis further validated the interactions between the peptides and the key hypertension-related targets identified through network pharmacology. Binding energy, an indirect measure of interaction strength, is considered favorable when the value is less than 0 kcal/mol, indicating spontaneous binding. Lower binding energy corresponds to greater conformational stability of the ligand–receptor complex [46]. In this study, all observed binding energies were negative, suggesting that the peptide–target interactions occurred spontaneously and were energetically favorable. Both peptides exhibited strong binding affinities toward REN, ACE, and AGTR2, consistent with predictions from the network pharmacology model. However, IVGRPLANG showed relatively weaker binding affinity toward CTSB and AGTR2, while IGDEPRHQYL exhibited the weakest interaction with AGTR2. The dominance of hydrogen bonding in the interaction profiles further supports the formation of stable and specific peptide–protein complexes, highlighting the potential of these venom-derived peptides as promising antihypertensive candidates.

## 4. Materials and Methods

### 4.1. Peptide Synthesis

In our previous study [26], the ACE-inhibitory peptides IVGRPLANG (896.48 Da) and IGDEPRHQYL (1227.65 Da) were identified using matrix-assisted laser desorption/ionization time-of-flight mass spectrometry (MALDI-TOF-MS). Based on these findings, both peptides were chemically synthesized by Peptron Co., Ltd. (Daejeon, Republic of Korea), with a confirmed purity of 95%.

### 4.2. ACE-Inhibitory Effect

The ACE-inhibitory activity of the peptides was assessed using the ACE Kit-WST (Dojindo Laboratories, Kumamoto, Japan) following the manufacturer’s protocol [47]. Both peptides were dissolved in distilled water, and serial dilutions were prepared at concentrations of 0.01, 0.10, 1, 10, 100, and 1000 µM. Captopril was used as the positive control. For the assay, 20 µL of each sample solution was added to the designated wells, including the sample, blank 1, and blank 2 wells. Subsequently, 20 µL of substrate buffer was added to all wells. Deionized water (20 µL) was added to blank 2 wells, while 20 µL of enzyme working solution was added to the sample and blank 1 wells. The plate was incubated at 37 °C for 1 h. Following incubation, 200 µL of indicator working solution was added to each well, and the plate was further incubated at room temperature for 10 min. Absorbance was measured at 450 nm using a microplate reader. ACE-inhibitory activity was calculated using the following equation:

ACE inhibition (%) = (Ablank1 − Asample)/(Ablank1 − Ablank2) × 100

where Ablank1 is the absorbance of the control (without inhibitor), Ablank2 is the absorbance of the reagent alone, and Asample is the absorbance of the peptide-treated sample.

### 4.3. Resistance to Simulated GI Digestion Assay

The stability of the synthesized peptides against gastrointestinal proteases was evaluated using a slightly modified method based on Dong et al. (2024) [32]. Peptide solutions at concentrations of 100 and 200 µM were sequentially subjected to enzymatic digestion. Initially, the peptides were incubated with pepsin (0.01%, pH 2.0) at 37 °C for 3 h. This was followed by a second digestion step with trypsin and chymotrypsin (each at 0.01%, pH 7.5) for an additional 3 h under the same temperature conditions. To terminate enzymatic activity, the reaction mixtures were boiled for 10 min. The samples were then centrifuged at 8000× *g* for 15 min, and the resulting supernatants were collected. The ACE-inhibitory activity of the digested peptide solutions was subsequently analyzed to assess their enzymatic stability.

### 4.4. Lineweaver–Burk Plot and Secondary Plot

The inhibition pattern of the peptides against ACE was analyzed using Lineweaver–Burk plots. ACE activity was measured at varying concentrations of each peptide (IGDEPRHQYL: 0, 5, and 10 µM; IVGRPLANG: 0, 20, and 40 µM) in the presence of different concentrations of the substrate hippuryl-histidyl-leucine (HHL), ranging from 0.625 to 20 mM. The Michaelis constant (Km) and maximum reaction velocity (Vmax) were calculated from the slope and y-intercept of the Lineweaver–Burk plots. Additionally, secondary plots were generated to determine the inhibition constant (Ki) values for each peptide based on the derived Km and Vmax values.

### 4.5. Molecular Dynamics Simulations

Molecular dynamics (MD) simulations were conducted for the complexes formed between the ACE protein (PDB ID:1O8A) and the two inhibitory peptides, IVGRPLANG and IGDEPRHQYL. Molecular dynamics (MD) simulations were conducted based on the following parameters:

- Software: Desmond (Schrödinger suite).
- Force field: OPLS-AA.
- Protein preparation: Protein–ligand complexes were optimized and refined using the Protein Preparation Wizard.
- System setup: All systems were solvated in an orthorhombic water box (125 Å × 125 Å × 125 Å) using the SPC water model. Systems were neutralized with appropriate counterions.
- Energy minimization: Performed using a combination of steepest descent (SD) and conjugate-gradient (CG) algorithms until convergence.
- Equilibration: Conducted using Desmond’s default relaxation protocol.
- Production MD:
  - Duration: 100 ns;
  - Ensemble: NPT (constant number of particles, pressure, temperature);
  - Temperature: 300 K;
  - Pressure: 1 atm;
  - Periodic boundary conditions applied in all directions;
  - Electrostatics calculated via Particle Mesh Ewald (PME);
  - Bond constraints: SHAKE algorithm used for hydrogen-containing bonds.
- Trajectory sampling: Saved at 10 ps intervals (0.01 ns).
- Analyses performed: RMSD, RMSF, Radius of Gyration (Rg), SASA, total energy (kcal/mol), and simulation event analysis.
- Initial structures: Obtained from top-ranked HADDOCK protein–peptide complexes based on docking score and cluster statistics. All trajectory analyses were carried out using the Desmond simulation package within the Schrödinger environment.

### 4.6. Peptide Mining Antihypertensive Targets

Putative peptide target sets were generated using SwissTargetPrediction and the Similarity Ensemble Approach (SEA) databases. Hypertension-related genes were retrieved from multiple sources, including GeneCards (https://www.genecards.org/, accessed on 11 February 2025), OMIM (https://omim.org/, accessed on 12 February 2025), PharmGKB (https://www.pharmgkb.org/, accessed on 13 February 2025), and DisGeNET (https://www.disgenet.com/, accessed on 14 February 2025). The collected genes were ranked according to their relevance scores to hypertension, and only those with a relatedness score greater than 2 were selected for further analysis. The intersection between predicted peptide targets and hypertension-related genes was identified using a Venn diagram. Subsequently, a protein–protein interaction (PPI) network was constructed using Cytoscape software (version 3.9.1) to visualize and analyze the functional relationships among the intersecting target proteins.

### 4.7. GO and KEGG Analysis

Gene Ontology (GO) functional annotation and Kyoto Encyclopedia of Genes and Genomes (KEGG) pathway enrichment analyses were performed using the STRING database (https://string-db.org/, accessed on 18 February 2025), with the organism set to Homo sapiens. GO analysis was categorized into three domains: biological processes (BPs), cellular components (CCs), and molecular functions (MFs). KEGG pathway enrichment analysis was conducted for target genes with a network degree score ≥10 to identify key signaling pathways potentially involved in the antihypertensive effects of the peptides.

### 4.8. Molecular Docking

Molecular docking studies for the two ACE-inhibitory peptides, IVGRPLANG and IGDEPRHQYL, were performed using HADDOCK version 2.4 (https://rascar.science.uu.nl/haddock2.4/, accessed on 27 February 2025). Protein–peptide docking was performed using the HADDOCK 2.4 web server. Input structures of the receptor (ACE protein) and peptide ligands were prepared in PDB format. Docking followed the standard three-stage HADDOCK protocol: (1) rigid-body energy minimization, (2) semi-flexible refinement, and (3) final refinement in an explicit solvent. The default parameters provided by HADDOCK were used for all docking stages unless otherwise specified. Active residues were defined based on prior literature and predicted interaction hotspots; passive residues were automatically selected by HADDOCK. The top-ranked docked complex was selected based on the HADDOCK score, which incorporates van der Waals, electrostatic, desolvation, and restraint violation energies, along with the cluster size and Z-score. The three-dimensional (3D) structures of the peptides were predicted using the Google ColabFold platform, with structural details previously reported by Prakash et al. (2024) [26]. These peptide structures were also utilized for docking with hypertension-related target proteins. The 3D structures of selected target proteins—such as Renin (REN) and Bradykinin Receptor B2 (BDKRB2)—were also generated using Google ColabFold and validated using AlphaFold’s predicted TM-score (pTM score). The available PDB structures for REN and BDKRB2 caused persistent errors in HADDOCK, even after attempted manual corrections. Additional target protein structures were retrieved from the Protein Data Bank (PDB), which is given in Table 3.

All receptor proteins were preprocessed using PyMOL v3.0, which included the removal of water molecules and co-crystallized ligands, followed by the addition of hydrogen atoms. The binding pockets of the target proteins were predicted using PrankWeb (https://prankweb.cz/, accessed on 25 February 2025), with detailed binding site coordinates listed in Table S2. Docked complexes were analyzed to visualize molecular interactions and binding orientations. Two-dimensional ligand–protein interaction diagrams were generated using PDBsum (https://www.ebi.ac.uk/thornton-srv/databases/pdbsum/, accessed on 23 February 2025). Docking scores and interaction metrics were obtained directly from HADDOCK version 2.4.

### 4.9. Statistical Analysis

All experiments were performed in triplicate, and data are expressed as mean ± standard deviation (SD). The statistical analysis was conducted using the nonparametric Kruskal-Walli’s test. Statistical significance levels were denoted as * *p* < 0.05. All analyses were performed using GraphPad Prism software (version 5.1).

## 5. Conclusions

This study comprehensively characterized two angiotensin-converting enzyme (ACE)-inhibitory peptides, IVGRPLANG and IGDEPRHQYL, derived from *Nemopilema nomurai* venom. Both peptides demonstrated competitive inhibition and strong binding affinity toward ACE. Molecular dynamics simulations confirmed the structural stability of the peptide–ACE complexes, while network pharmacology analysis identified several hypertension-related targets, particularly those associated with the renin–angiotensin–aldosterone system (RAAS), underscoring the peptides’ broader therapeutic potential. Molecular docking further validated their interactions with key targets, supporting their candidacy as promising multitarget antihypertensive agents. Future studies will focus on elucidating the in vivo efficacy and underlying molecular mechanisms of these venom-derived ACE inhibitors. Specifically, pharmacological targets associated with ACE inhibition—such as AGTR2, REN, and BDKRB2—identified through network pharmacology analysis, will be experimentally validated using Western blotting and/or real-time PCR.

## Figures and Tables

**Table 1:** Kinetic parameters and inhibition mode of ACE by synthetic peptides IVGRPLANG and IGDEPRHQYL.

Peptide	Concentration (µM)	Vmax	Km	Ki (µM)	Mode of Inhibition
IVGRPLANG	0	1.919 ± 0.00019	3.145 ± 0.0196	51.389	-
	20	1.918 ± 0.00021	3.982 ± 0.00383		Competitive
	40	1.811 ± 0.00076	5.175 ± 0.02686		Competitive
IGDEPRHQYL	0	1.919 ± 0.00019	3.147 ± 0.0196	5.457	-
	5	1.953 ± 0.00613	5.801 ± 0.10923		Competitive
	10	2.015 ± 0.05609	9.198 ± 0.39909		Competitive

**Table 2:** Docking and clustering scores of potential key antihypertensive targets with peptides IVGRPLANG and IGDEPRHQYL.

Targets for IVGRPLANG	Cluster Number	Cluster Size ^a^	HADDOCK Score ^b^	Overall RMSD ^c^	Z-Score ^d^
Renin	4	15	−65.4 ± 2.5	2.3 ± 0.0	−1.2
Cathepsin S	1	42	−54.5 ± 4.1	0.3 ± 0.2	−1.9
Cathepsin B	8	7	−27.8 ± 6.2	2.8 ± 0.0	−2.2
Angiotensin-Converting Enzyme	1	20	−17.2 ± 5.1	1.5 ± 0.0	−2.1
Angiotensin II Receptor type 2	3	18	−29.5 ± 5.3	0.4 ± 0.3	−2.5
**Targets for** **IGDEPRHQYL**					
Angiotensin	3	18	−72.2 ± 1.8	2.2 ± 0.1	−1.3
Angiotensin II Receptor type 1	3	12	−60.3 ± 3.2	3.0 ± 0.0	−1.7
Angiotensin II Receptor type 2	3	12	−36.3 ± 4.0	0.8 ± 0.2	−2.9
Renin	2	42	−67.3 ± 3.5	2.2 ± 0.2	−1
Bradykinin receptor 2	1	36	−87.4 ± 2.8	2.7 ± 0.1	−1.9
Kininogen I	2	44	−115.3 ± 8.3	0.3 ± 0.2	−1.7

^a^ The analysis is based on a total of 200 generated models. ^b^ The best HADDOCK score clusters are reported according to the lowest energy score, which is the sum of Van der Waals, electrostatic, desolvation, and restraint energies, averaged over the top four structures within the cluster. ^c^ RMSD is calculated between the four best structures within the cluster. ^d^ The Z-score is determined by calculating the standard deviation of the HADDOCK score for each cluster, relative to the mean of all clusters.

**Table 3:** Protein and gene information with corresponding PDB IDs.

Protein Name	Gene Symbol	PDB ID
Angiotensinogen	AGT	2WXW
Angiotensin Receptor 1	AGTR1	4YAY
Angiotensin Receptor 2	AGTR2	5UNF
Kininogen 1	KNG1	1NY2
Angiotensin-Converting Enzyme	ACE	1O8A
Cathepsin S	CTSS	4P6G
Cathepsin B	CTSB	1GMY

## Data Availability

The original contributions presented in the study are included in the article/Supplementary Materials; further inquiries can be directed to the corresponding author/s.

## Supplementary Materials

The following supporting information can be downloaded at https://www.mdpi.com/article/10.3390/md23070267/s1, Figure S1. Prediction of the protein BDKRB2 structures by ColabFold and models were ranked based on AlphaFold pTM Score. Figure S2. Prediction of the protein RENIN structures by ColabFold and models were ranked based on AlphaFold pTM Score. Table S1. Protein Structural Scoring for modeled protein using ColabFold and AlphaFold. Table S2. Binding pockets obtained from Prank Web (https://prankweb.cz/) online software for hypertensive target proteins. Table S3. HADDOCK scores for hypertensive target proteins against IVGRPLANG ACE-inhibitory peptide. Table S4. HADDOCK scores for hypertensive target proteins against IGDEPRHQYL ACE-inhibitory peptide.
