# Supplementary material for: Characterization of Novel ACE-Inhibitory Peptides from Nemopilema nomurai Jellyfish Venom Hydrolysate: In Vitro and In Silico Approaches
Source: Mar Drugs. 2025 Jun 26;23(7):267. doi: 10.3390/md23070267 (PMC12298423; doi:10.3390/md23070267)
Supplement: Supplementary file 1 [file marinedrugs-23-00267-s001.zip › marinedrugs-3697218-supplementary.pdf]

Characterization of Novel ACE-Inhibitory Peptides from *Nemopilema nomurai* Jellyfish Venom hydrolysate: *In vitro* and *In silico* Approaches

Supplementary file

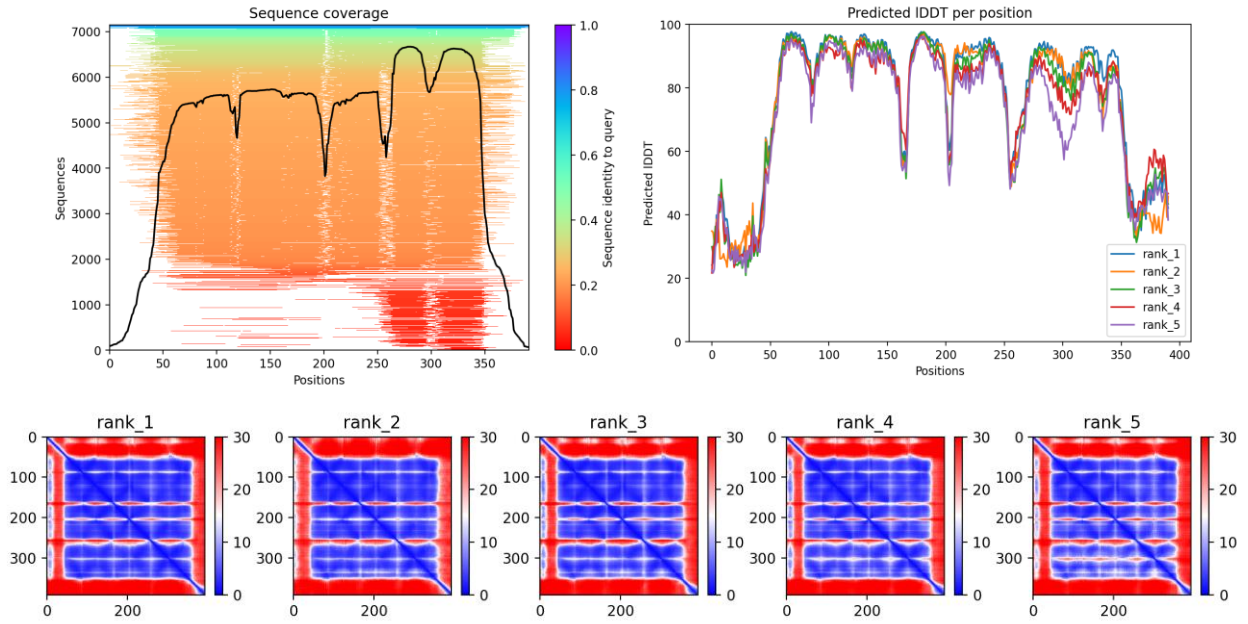

Figure S1. Prediction of the protein BDKRB2 structures by ColabFold and models were ranked based on AlphaFold pTM Score.

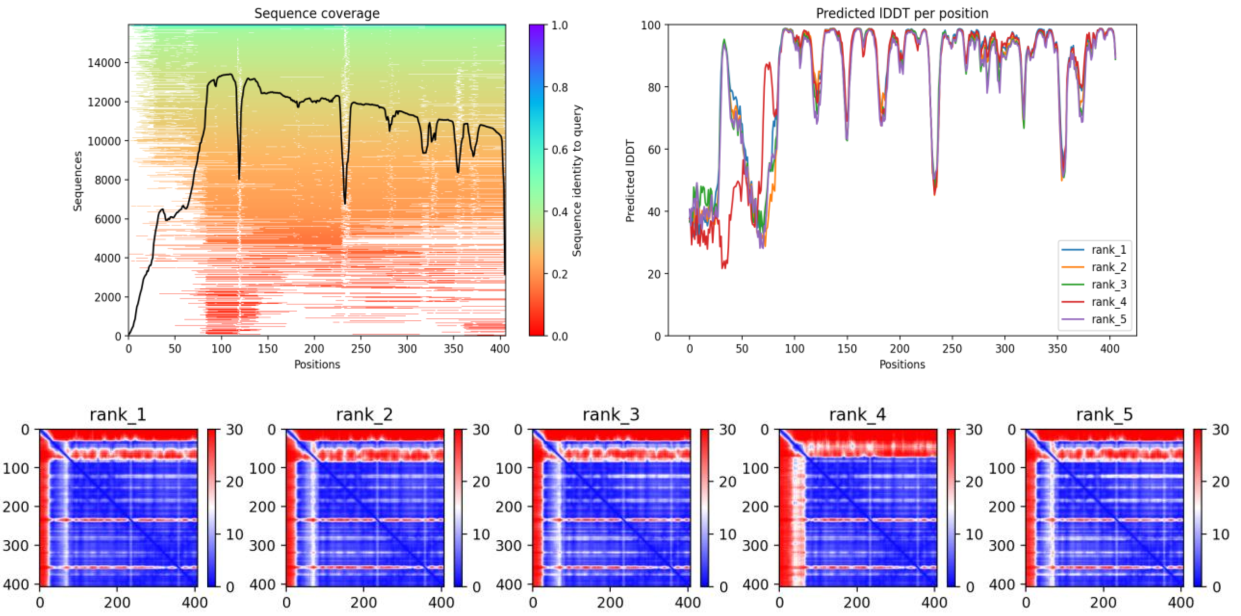

Figure S2. Prediction of the protein RENIN structures by ColabFold and models were ranked based on AlphaFold pTM Score.

Table S1. Protein Structural Scoring for modeled protein using ColabFold and AlphaFold

Protein Name	Score	Model 1	Model 2	Model 3	Model 4	Model 5
Bradykinin Potential Peptide Receptor 2	pLDDT	74.6	72.3	78.1	75.5	76.2
	pTM	0.726	0.701	0.729	0.722	0.731
Renin	pLDDT	78.4	77	79.6	82.1	79.6
	pTM	0.771	0.767	0.783	0.787	0.794

Table S2. Binding pockets obtained from Prank Web online software for hypertensive target proteins.

Protein Symbol	Score	Center X	Center Y	Center Z	Residue id's
**KNG1**	24.39	113.3407	102.9115	165.156	B_387 B_388 B_391 C_582 C_583 C_584 C_585 C_586 C_588 C_624 C_625 C_626 C_628 C_670 C_671 C_673 C_674 C_711 C_712 C_713 C_714 C_715 C_753 C_754 C_755 C_756 C_757 C_758 C_759 C_769 C_797 C_798 C_799 C_800 C_801 C_802 C_813 C_837 C_839 C_840 C_841 C_842 C_843 C_844 C_845 C_855
**ACE2**	29.47	134.3511	129.6114	208.9938	B_152 B_156 B_161 B_169 B_237 B_240 B_241 B_244 B_248 B_251 B_252 B_262 B_263 B_264 B_266 B_267 B_268 B_270 B_271 B_272 B_275 B_277 B_278 B_280 B_281 B_282 B_452 B_477 B_481 B_485 B_487 B_499 B_606
**AGT**	5.94	4.6624	-66.4439	-6.2334	A_114 A_115 A_117 A_118 A_119 A_122 A_13 A_138 A_141 A_142 A_143 A_144 A_145 A_18 A_21 A_22 A_25 A_380
**AGTR1**	13.69	-18.1434	13.6016	39.559	A_105 A_108 A_167 A_179 A_180 A_21 A_23 A_281 A_284 A_285 A_288 A_289 A_292 A_31 A_35 A_77 A_84 A_87 A_88 A_92
**AGTR2**	75.57	72.8438	5.6651	22.3808	A_133 A_136 A_137 A_140 A_141 A_143 A_144 A_163 A_166 A_167 A_170 A_173 A_174 A_177 A_180 A_181 A_198 A_200 A_203 A_206 A_207 A_210 A_211 A_214 A_217 A_218 A_222 A_225 A_226 A_229 A_230 A_233 A_236 A_82 B_115 B_116 B_118 B_119 B_120 B_122 B_123 B_126 B_127 B_158 B_160 B_161 B_164 B_165 B_168 B_172 B_175 B_176 B_179 B_69 B_72 B_77 B_78 B_80 B_81 B_84 B_85 B_88 B_92
**BDKRB2**	18	-11.4027	2.6984	11.7754	A_148 A_151 A_152 A_155 A_264 A_265 A_267 A_268 A_271 A_272 A_275 A_332 A_335 A_336 A_337 A_338 A_339 A_82 A_90 A_92 A_93 A_96

**REN**	20.25	-0.4393	9.516	11.534	150, 151, 183, 295, 296, 297, 299, 302, 316, 318, 319, 320, 321, 367, 368, 369, 380, 43, 59, 60, 61, 63, 64, 78, 80, 82
**ACE**	100.62	41.5132	33.2511	46.7924	124, 136, 139, 140, 143, 146, 161, 162, 166, 169, 276, 277, 278, 279, 281, 282, 284, 285, 351, 353, 354, 355, 356, 357, 358, 360, 369, 370, 372, 374, 376, 377, 379, 380, 383, 384, 387, 391, 399, 402, 403, 407, 410, 411, 415, 453, 454, 457, 511, 512, 513, 516, 518, 519, 520, 522, 523, 527, 59, 62, 63, 66, 69, 70, 81, 82, 85
**CTSB**	21.41	43.6302	34.1596	25.698	174, 175, 177, 195, 198, 245, 110, 111, 119, 120, 122, 173, 174, 175, 176, 181, 196, 197, 198, 199, 200, 23, 24, 26, 27, 29, 68, 69, 72, 73, 74, 75, 76
**CTSS**	59.88	11.4196	14.2324	98.768	113, 115, 133, 136, 142, 157, 158, 159, 160, 177, 18, 19, 191, 20, 205, 23, 25, 63, 64, 65, 66, 67, 68, 69, 115, 116, 133, 136, 142, 157, 158, 159, 160, 177, 18, 19, 190, 191, 20, 204, 205, 206, 207, 21, 23, 25, 26, 63, 64, 65, 66, 67, 68, 69

Table S3. HADDOCK scores for hypertensive target proteins against IVGRPLANG ACE inhibitory peptide.

**(AGTR2) Angiotensin receptor 2**	**Cluster 3**	**Cluster 7**	**Cluster 6**	**Cluster 17**	**Cluster 11**	**Cluster 1**	**Cluster 8**	**Cluster 4**	**Cluster 2**	**Cluster 9**
HADDOCK score	-29.5 ± 5.3	-14.2 ± 4.3	-4.0 ± 13.7	-2.5 ± 11.4	0.6 ± 5.1	0.7 ± 5.7	5.2 ± 9.4	6.8 ± 8.9	7.1 ± 6.1	7.5 ± 9.5
Cluster size	18	6	6	4	5	21	5	11	18	5
RMSD from the overall lowest-energy structure	0.4 ± 0.3	0.8 ± 0.1	2.6 ± 0.1	0.8 ± 0.0	0.6 ± 0.1	2.9 ± 0.1	2.9 ± 0.0	2.4 ± 0.1	2.8 ± 0.0	2.6 ± 0.0
Van der Waals energy	-43.0 ± 3.4	-51.2 ± 3.9	-40.0 ± 4.7	-35.5 ± 5.0	-37.7 ± 4.1	-33.4 ± 4.1	-33.4 ± 4.0	-35.5 ± 2.5	-32.8 ± 2.5	-39.3 ± 2.5
Electrostatic energy	-176.4 ± 20.8	-79.8 ± 15.0	-21.6 ± 9.3	-144.2 ± 29.1	-134.2 ± 24.3	-8.5 ± 1.9	-33.9 ± 7.5	-22.1 ± 10.6	-12.2 ± 5.5	-29.7 ± 8.1
Desolvation energy	-10.6 ± 1.1	-18.3 ± 1.4	-18.5 ± 1.7	-7.0 ± 1.5	-5.9 ± 1.4	-17.7 ± 1.8	-16.5 ± 1.0	-14.1 ± 2.1	-15.4 ± 1.9	-15.0 ± 1.2
Restraints violating energy	594.8 ± 27.0	712.6 ± 72.9	588.4 ± 134.5	688.5 ± 72.3	710.0 ± 38.5	535.5 ± 100.2	619.9 ± 54.1	608.6 ± 49.7	577.5 ± 76.9	677.8 ± 77.8
Buried Surface Area	1667.8 ± 32.4	1666.3 ± 52.0	1249.4 ± 44.8	1525.0 ± 81.7	1568.2 ± 42.9	1134.2 ± 97.6	1195.8 ± 36.2	1159.4 ± 53.2	1127.1 ± 43.9	1198.3 ± 6.4
Z-Score	-2.5	-1.1	-0.2	0	0.3	0.3	0.7	0.8	0.8	0.9
**(ACE) Angiotensin Converting Enzyme**	**Cluster 1**	**Cluster 5**	**Cluster 2**	**Cluster 3**	**Cluster 7**	**Cluster 11**	**Cluster 9**	**Cluster 13**	**Cluster 6**	**Cluster 10**
HADDOCK score	-17.2 ± 5.1	0.6 ± 3.8	0.9 ± 2.7	2.9 ± 5.2	3.1 ± 3.8	3.2 ± 12.5	8.3 ± 9.2	13.1 ± 20.8	21.3 ± 6.0	24.9 ± 8.2
Cluster size	20	12	15	14	8	5	6	4	11	5
RMSD from the overall lowest-energy structure	1.5 ± 0.0	1.9 ± 0.0	1.9 ± 0.1	1.3 ± 0.1	1.7 ± 0.0	1.7 ± 0.0	2.2 ± 0.0	1.8 ± 0.1	1.5 ± 0.2	1.8 ± 0.0
Van der Waals energy	-47.1 ± 3.9	-36.0 ± 4.2	-39.4 ± 5.0	-23.8 ± 2.3	-34.1 ± 2.1	-43.0 ± 5.5	-36.9 ± 3.1	-38.6 ± 8.9	-32.1 ± 3.3	-40.2 ± 4.5
Electrostatic energy	-172.4 ± 15.5	-98.0 ± 23.5	-143.3 ± 13.2	-118.3 ± 9.3	-158.3 ± 13.3	-138.4 ± 18.9	-79.8 ± 20.0	-112.7 ± 10.9	-117.8 ± 19.1	-78.5 ± 39.2
Desolvation energy	4.1 ± 1.4	-1.7 ± 2.1	3.6 ± 1.2	1.6 ± 1.2	1.8 ± 0.2	1.1 ± 1.0	-3.9 ± 0.8	0.3 ± 1.5	2.9 ± 1.7	2.5 ± 2.9
Restraints violation energy	602.9 ± 37.6	579.0 ± 42.5	654.3 ± 42.4	486.9 ± 59.0	670.5 ± 28.9	727.7 ± 71.6	649.9 ± 90.1	740.3 ± 124.8	740.9 ± 64.3	782.7 ± 57.6

Buried Surface Area	1476.4 ± 40.0	1434.5 ± 33.9	1440.6 ± 87.4	1285.8 ± 47.3	1303.8 ± 23.1	1350.1 ± 39.3	1223.8 ± 49.9	1420.3 ± 142.0	1247.8 ± 50.8	1250.4 ± 46.3
Z-Score	-2.1	-0.5	-0.5	-0.3	-0.3	-0.3	0.2	0.6	1.3	1.7
**(CTSS) Cathespin S**	**Cluster 1**	**Cluster 3**	**Cluster 2**	**Cluster 7**	**Cluster 12**	**Cluster 4**	**Cluster 5**	**Cluster 11**	**Cluster 8**	**Cluster 6**
HADDOCK score	-54.5 ± 4.1	-48.8 ± 4.5	-47.4 ± 3.5	-44.5 ± 7.0	-38.9 ± 2.6	-35.3 ± 5.1	-34.0 ± 4.5	-33.3 ± 6.7	-33.2 ± 11.0	-30.7 ± 2.2
Cluster size	42	26	29	9	5	15	10	5	7	9
RMSD from the overall lowest-energy structure	0.3 ± 0.2	1.5 ± 0.1	3.6 ± 0.2	1.5 ± 0.0	1.3 ± 0.1	1.4 ± 0.0	3.3 ± 0.1	3.6 ± 0.1	3.9 ± 0.1	2.5 ± 0.1
Van der Waals energy	-63.3 ± 4.3	-51.8 ± 2.3	-52.2 ± 1.2	-56.9 ± 2.4	-55.3 ± 2.4	-43.8 ± 4.4	-43.2 ± 7.8	-44.9 ± 6.6	-39.2 ± 1.6	-42.0 ± 1.4
Electrostatic energy	-56.3 ± 18.2	-64.8 ± 7.6	-91.9 ± 29.6	-74.4 ± 11.0	-57.2 ± 5.3	-81.3 ± 24.1	-91.3 ± 14.6	-76.0 ± 16.5	-98.0 ± 10.5	-68.9 ± 9.2
Desolvation energy	-14.0 ± 2.9	-10.8 ± 1.8	-8.8 ± 2.0	-7.8 ± 3.3	-8.0 ± 3.0	-10.7 ± 0.8	-5.1 ± 3.0	-10.4 ± 2.7	-9.9 ± 2.5	-4.3 ± 1.7
Restraints violation energy	340.5 ± 58.9	268.6 ± 61.3	320.2 ± 31.1	350.6 ± 44.6	357.8 ± 42.4	354.3 ± 44.7	325.3 ± 27.1	371.9 ± 44.4	354.9 ± 85.8	293.8 ± 11.4
Buried Surface Area	1412.7 ± 45.5	1368.5 ± 54.8	1330.2 ± 25.6	1285.4 ± 57.9	1387.9 ± 31.6	1292.6 ± 35.6	1328.8 ± 42.1	1321.3 ± 26.9	1214.6 ± 39.5	1260.6 ± 24.0
Z-Score	-1.9	-1.1	-0.9	-0.6	0.2	0.6	0.8	0.9	0.9	1.2
**(CTSB) Cathespin B**	**Cluster 8**	**Cluster 5**	**Cluster 4**	**Cluster 3**	**Cluster 14**	**Cluster 13**	**Cluster 2**	**Cluster 1**	**Cluster 11**	**Cluster 16**
HADDOCK score	-27.8 ± 6.2	-20.5 ± 6.0	-19.9 ± 8.7	-19.1 ± 7.1	-17.6 ± 3.6	-16.4 ± 5.9	-14.8 ± 2.1	-12.9 ± 3.6	-11.6 ± 4.5	-9.9 ± 10.3
Cluster size	7	13	14	15	5	5	16	18	6	4
RMSD from the overall lowest-energy structure	2.8 ± 0.0	4.0 ± 0.1	4.7 ± 0.1	3.1 ± 0.2	3.8 ± 0.1	4.5 ± 0.0	4.9 ± 0.1	3.9 ± 0.1	4.6 ± 0.1	2.3 ± 0.1
Van der Waals energy	-27.4 ± 7.6	-36.9 ± 4.2	-42.4 ± 8.5	-28.8 ± 4.2	-32.6 ± 11.9	-35.0 ± 4.2	-29.6 ± 4.7	-36.8 ± 6.7	-43.1 ± 2.5	-32.7 ± 2.0
Electrostatic energy	-154.4 ± 21.3	-83.5 ± 10.8	-90.0 ± 35.3	-135.1 ± 38.0	-122.3 ± 12.3	-124.8 ± 26.7	-110.2 ± 8.9	-77.4 ± 34.7	-104.4 ± 27.6	-107.7 ± 7.4
Desolvation energy	1.9 ± 1.8	0.9 ± 1.8	-1.8 ± 1.3	2.3 ± 0.9	7.1 ± 3.3	1.4 ± 1.2	1.1 ± 0.5	-0.4 ± 3.0	1.7 ± 1.3	2.7 ± 0.4

Restraints violation energy	286.4 ± 104.9	322.8 ± 71.4	423.2 ± 53.5	344.2 ± 73.6	323.5 ± 84.8	421.6 ± 63.1	358.0 ± 51.3	398.3 ± 62.0	506.4 ± 26.5	416.1 ± 82.1
Buried Surface Area	1072.0 ± 110.3	1152.8 ± 31.4	1221.2 ± 13.0	1070.1 ± 32.8	1074.2 ± 147.1	1188.0 ± 6.8	1012.5 ± 82.0	1100.8 ± 82.8	1142.4 ± 10.2	1177.8 ± 55.4
Z-Score	-2.2	-0.7	-0.6	-0.4	-0.1	0.1	0.5	0.8	1.1	1.4
**(REN) Renin**	**Cluster 4**	**Cluster 2**	**Cluster 1**	**Cluster 3**	**Cluster 6**	**Cluster 7**	**Cluster 5**			
HADDOCK score	-65.4 ± 2.5	-64.5 ± 4.0	-60.7 ± 1.4	-51.4 ± 2.7	-40.5 ± 5.7	-37.6 ± 3.6	-37.4 ± 4.0			
Cluster size	15	43	84	37	4	4	4			
RMSD from the overall lowest-energy structure	2.3 ± 0.0	0.3 ± 0.2	1.7 ± 0.0	2.3 ± 0.1	2.3 ± 0.0	2.4 ± 0.0	2.2 ± 0.0			
Van der Waals energy	-57.0 ± 0.9	-54.4 ± 3.9	-43.8 ± 1.4	-38.5 ± 4.9	-37.5 ± 3.1	-33.1 ± 2.1	-34.8 ± 2.0			
Electrostatic energy	-34.8 ± 6.2	-44.5 ± 11.8	-109.0 ± 6.0	-71.6 ± 20.1	-29.9 ± 8.5	-49.9 ± 30.9	-20.9 ± 9.2			
Desolvation energy	-6.0 ± 0.5	-4.4 ± 2.1	-0.5 ± 0.5	-2.5 ± 1.5	-5.0 ± 0.4	-4.1 ± 3.1	-7.0 ± 1.5			
Restraints violation energy	45.6 ± 25.5	31.8 ± 8.3	53.7 ± 17.0	38.7 ± 36.3	79.2 ± 25.5	95.1 ± 14.7	85.7 ± 17.4			
Buried Surface Area	1351.7 ± 21.8	1299.0 ± 45.1	1278.7 ± 21.0	1158.7 ± 47.1	1160.6 ± 30.4	1110.9 ± 51.9	960.5 ± 33.0			
Z-Score	-1.2	-1.1	-0.8	0	0.9	1.1	1.2			

Table S4. HADDOCK scores for hypertensive target proteins against IGDEPRHQYL ACE inhibitory peptide.

**AGT (Angiotensin)**	**Cluster 3**	**Cluster 1**	**Cluster 5**	**Cluster 7**	**Cluster 2**	**Cluster 10**	**Cluster 6**	**Cluster 4**	**Cluster 8**	**Cluster 9**
HADDOCK score	-72.2 ± 1.8	-68.5 ± 4.5	-68.5 ± 2.3	-66.8 ± 8.1	-61.4 ± 9.0	-52.0 ± 8.2	-50.6 ± 3.6	-42.6 ± 1.9	-38.8 ± 9.1	-38.2 ± 9.8
Cluster size	18	42	11	9	26	4	9	12	5	4
RMSD from the overall lowest-energy structure	2.2 ± 0.1	1.6 ± 0.0	2.4 ± 0.0	1.5 ± 0.0	2.6 ± 0.1	1.3 ± 0.2	1.4 ± 0.2	2.2 ± 0.1	2.4 ± 0.1	2.0 ± 0.1
Van der Waals energy	-37.6 ± 4.2	-38.5 ± 3.0	-32.7 ± 3.9	-35.3 ± 7.1	-30.0 ± 8.3	-28.6 ± 7.1	-32.1 ± 3.9	-15.8 ± 3.8	-22.7 ± 6.5	-25.7 ± 6.0
Electrostatic energy	-174.9 ± 23.9	-183.3 ± 16.9	-196.9 ± 21.2	-205.0 ± 37.2	-219.1 ± 30.8	-191.5 ± 16.6	-117.2 ± 29.9	-151.7 ± 38.1	-136.7 ± 28.9	-99.9 ± 18.7
Desolvation energy	-4.8 ± 2.8	1.9 ± 1.9	-0.2 ± 2.4	-1.2 ± 3.3	4.8 ± 2.4	6.4 ± 1.3	-2.8 ± 5.6	-4.3 ± 1.0	3.0 ± 4.4	4.3 ± 1.5
Restraints violation energy	52.2 ± 11.0	48.3 ± 29.9	38.7 ± 19.1	106.4 ± 25.4	76.0 ± 29.7	84.4 ± 15.7	77.7 ± 36.2	78.6 ± 42.1	81.9 ± 11.7	31.4 ± 19.2
Buried Surface Area	1314.2 ± 50.8	1263.8 ± 43.2	1174.8 ± 78.4	1344.7 ± 55.5	1292.8 ± 144.9	1214.4 ± 109.9	1134.9 ± 115.2	843.9 ± 61.9	956.2 ± 109.1	877.5 ± 62.7
Z-Score	-1.3	-1	-1	-0.9	-0.4	0.3	0.4	1.1	1.4	1.4
**AGTR1 (Angiotensin receptor 1)**	**Cluster 3**	**Cluster 1**	**Cluster 13**	**Cluster 7**	**Cluster 5**	**Cluster 6**	**Cluster 2**	**Cluster 4**	**Cluster 12**	**Cluster 8**
HADDOCK score	-60.3 ± 3.2	-53.6 ± 2.0	-53.6 ± 10.9	-52.3 ± 6.6	-49.8 ± 5.0	-45.5 ± 8.5	-43.0 ± 4.6	-40.9 ± 3.9	-36.4 ± 8.6	-36.1 ± 4.1
Cluster size	12	14	4	7	7	7	13	10	4	6
RMSD from the overall lowest-energy structure	3.0 ± 0.0	2.0 ± 0.2	2.3 ± 0.1	2.6 ± 0.1	2.2 ± 0.2	4.2 ± 0.1	3.5 ± 0.2	1.6 ± 0.3	3.2 ± 0.3	3.2 ± 0.0
Van der Waals energy	-36.9 ± 3.1	-27.1 ± 5.5	-35.5 ± 7.1	-31.8 ± 2.1	-31.3 ± 4.8	-38.0 ± 3.3	-36.8 ± 1.2	-17.7 ± 5.1	-24.1 ± 1.9	-24.8 ± 5.3
Electrostatic energy	-173.8 ± 7.2	-204.1 ± 10.7	-155.9 ± 17.6	-145.0 ± 26.1	-155.8 ± 19.9	-39.1 ± 6.5	-57.8 ± 16.7	-204.7 ± 34.8	-77.1 ± 33.0	-150.7 ± 17.4
Desolvation energy	-0.8 ± 1.8	-8.3 ± 1.6	4.4 ± 2.0	-4.0 ± 1.6	-3.0 ± 3.0	-22.0 ± 3.2	-13.1 ± 6.1	-3.1 ± 3.1	-19.9 ± 2.4	-2.6 ± 2.5

Restraints violation energy	121.3 ± 52.5	226.7 ± 54.3	86.9 ± 42.6	124.9 ± 35.6	156.5 ± 27.1	223.1 ± 35.5	184.4 ± 34.2	208.9 ± 33.3	229.6 ± 31.5	214.5 ± 12.9
Buried Surface Area	1064.8 ± 11.7	940.1 ± 44.1	1159.7 ± 116.7	998.8 ± 70.3	930.4 ± 50.6	1039.7 ± 71.0	1065.7 ± 46.8	923.0 ± 65.6	815.5 ± 66.8	842.0 ± 27.9
Z-Score	-1.7	-0.8	-0.8	-0.7	-0.4	0.2	0.5	0.8	1.4	1.4
**AGTR2 (Angiotensin receptor 2)**	**Cluster 3**	**Cluster 12**	**Cluster 17**	**Cluster 1**	**Cluster 2**	**Cluster 16**	**Cluster 5**	**Cluster 4**	**Cluster 14**	**Cluster 15**
HADDOCK score	-36.3 ± 4.0	-13.3 ± 8.7	-8.8 ± 11.8	-8.8 ± 3.8	-8.7 ± 9.7	-7.9 ± 8.2	-7.7 ± 6.9	-7.6 ± 3.6	-6.3 ± 5.6	-3.4 ± 5.4
Cluster size	12	4	5	19	15	4	8	11	5	4
RMSD from the overall lowest-energy structure	0.8 ± 0.2	3.5 ± 0.1	3.2 ± 0.1	3.0 ± 0.1	2.7 ± 0.1	3.6 ± 0.0	3.3 ± 0.1	2.9 ± 0.1	3.3 ± 0.0	3.1 ± 0.1
Van der Waals energy	-51.5 ± 3.0	-33.6 ± 3.8	-39.9 ± 10.7	-40.7 ± 5.1	-44.3 ± 4.8	-48.2 ± 2.5	-43.9 ± 3.3	-48.5 ± 3.9	-54.0 ± 2.9	-42.7 ± 5.5
Electrostatic energy	-221.8 ± 30.2	-73.6 ± 43.4	-41.5 ± 11.0	-8.5 ± 3.6	-9.3 ± 7.0	-25.4 ± 3.1	-45.4 ± 21.3	-17.3 ± 10.4	-63.7 ± 10.9	-13.6 ± 4.6
Desolvation energy	-10.4 ± 1.2	-24.6 ± 1.6	-25.1 ± 4.0	-26.7 ± 3.1	-24.7 ± 1.7	-36.6 ± 4.3	-21.4 ± 5.2	-23.3 ± 4.6	-20.9 ± 2.9	-22.7 ± 3.4
Restraints violation energy	698.7 ± 34.6	596.3 ± 45.1	645.2 ± 39.9	603.7 ± 66.8	621.8 ± 125.6	820.5 ± 37.6	665.9 ± 75.6	676.0 ± 63.8	813.5 ± 25.5	647.0 ± 60.2
Buried Surface Area	1671.0 ± 89.6	1336.5 ± 50.6	1329.3 ± 83.8	1294.7 ± 76.8	1181.7 ± 52.2	1297.8 ± 62.1	1412.3 ± 30.2	1384.5 ± 68.3	1368.8 ± 34.2	1393.8 ± 73.2
Z-Score	-2.9	-0.3	0.2	0.2	0.2	0.3	0.4	0.4	0.5	0.9
**(ACE2) Angiotensin converting enzyme 2**	**Cluster 3**	**Cluster 11**	**Cluster 2**	**Cluster 1**	**Cluster 9**	**Cluster 6**	**Cluster 10**	**Cluster 8**	**Cluster 5**	**Cluster 4**
HADDOCK score	-51.0 ± 7.6	-49.0 ± 9.4	-37.0 ± 3.1	-34.2 ± 3.1	-33.3 ± 8.1	-31.6 ± 7.6	-28.9 ± 8.3	-22.2 ± 11.2	-19.1 ± 7.6	-15.4 ± 13.0
Cluster size	9	4	12	24	4	5	4	4	7	8
RMSD from the overall lowest-energy structure	0.6 ± 0.4	2.4 ± 0.1	1.6 ± 0.0	0.8 ± 0.1	0.8 ± 0.1	1.2 ± 0.2	2.7 ± 0.0	0.9 ± 0.1	1.0 ± 0.1	1.7 ± 0.1
Van der Waals energy	-55.7 ± 4.1	-48.7 ± 11.4	-55.3 ± 5.8	-48.4 ± 2.7	-44.3 ± 4.4	-41.8 ± 3.4	-34.5 ± 6.4	-47.2 ± 5.0	-38.5 ± 3.9	-38.6 ± 6.0

Electrostatic energy	-15.2 ± 3.3	-163.7 ± 25.5	-26.1 ± 3.7	-45.9 ± 19.6	-76.5 ± 24.1	-37.4 ± 9.9	-203.4 ± 52.1	-32.2 ± 2.6	-31.8 ± 11.4	-9.6 ± 6.9
Desolvation energy	-41.5 ± 1.0	-9.6 ± 3.9	-23.6 ± 3.3	-17.5 ± 3.1	-20.4 ± 1.3	-20.6 ± 2.5	4.4 ± 4.7	-20.3 ± 4.8	-28.6 ± 2.7	-20.0 ± 3.0
Restraints violation energy	492.4 ± 85.4	420.4 ± 56.7	470.0 ± 62.6	409.3 ± 24.2	467.1 ± 74.3	383.6 ± 77.9	418.5 ± 66.8	517.6 ± 81.1	542.8 ± 99.3	450.9 ± 97.7
Buried Surface Area	1595.0 ± 98.4	1622.2 ± 163.5	1499.5 ± 13.9	1406.1 ± 90.9	1309.4 ± 50.0	1252.8 ± 40.3	1347.0 ± 154.0	1325.1 ± 97.5	1257.9 ± 40.3	1210.0 ± 140.3
Z-Score	-1.7	-1.5	-0.4	-0.2	-0.1	0.1	0.3	0.9	1.2	1.5
**(KNG-1) Kininogen-1**	**Cluster 2**	**Cluster 1**	**Cluster 4**	**Cluster 3**						
HADDOCK score	-115.3 ± 8.3	-78.5 ± 3.3	-75.2 ± 4.3	-73.5 ± 1.6						
Cluster size	44	119	4	25						
RMSD from the overall lowest-energy structure	0.3 ± 0.2	1.9 ± 0.0	1.9 ± 0.0	2.0 ± 0.0						
Van der Waals energy	-67.4 ± 5.2	-62.7 ± 5.0	-62.8 ± 5.2	-50.8 ± 2.6						
Electrostatic energy	-257.7 ± 20.0	-109.9 ± 31.1	-112.3 ± 6.4	-207.8 ± 19.2						
Desolvation energy	-1.0 ± 2.2	-9.3 ± 1.0	-8.9 ± 2.4	-8.0 ± 1.6						
Restraints violation energy	46.1 ± 20.4	154.7 ± 31.9	188.8 ± 23.7	267.6 ± 17.3						
Buried Surface Area	1904.0 ± 48.7	1716.0 ± 12.8	1719.7 ± 15.1	1657.8 ± 62.6						
Z-Score	-1.7	0.4	0.6	0.7						
**(BRKB2) B2 bradykinin receptor**	**Cluster 1**	**Cluster 9**	**Cluster 4**	**Cluster 5**	**Cluster 13**	**Cluster 2**	**Cluster 6**	**Cluster 7**	**Cluster 8**	**Cluster 11**
HADDOCK score	-87.4 ± 2.8	-79.3 ± 8.2	-72.4 ± 1.2	-72.1 ± 3.4	-67.2 ± 4.4	-66.3 ± 0.6	-64.2 ± 1.6	-56.3 ± 6.8	-54.4 ± 11.7	-53.4 ± 15.8
Cluster size	36	5	11	10	4	23	10	9	5	4
RMSD from the overall lowest-energy structure	2.7 ± 0.1	0.4 ± 0.2	3.0 ± 0.0	2.9 ± 0.0	2.2 ± 0.1	1.1 ± 0.1	3.4 ± 0.1	2.3 ± 0.1	3.6 ± 0.1	3.5 ± 0.2

Van der Waals energy	-33.1 ± 4.8	-29.7 ± 3.5	-23.9 ± 8.7	-28.0 ± 3.9	-29.0 ± 7.7	-32.2 ± 7.7	-26.9 ± 7.6	-13.9 ± 5.2	-15.1 ± 5.1	-22.1 ± 6.6
Electrostatic energy	-324.9 ± 31.8	-304.3 ± 21.6	-312.4 ± 42.5	-234.4 ± 13.1	-220.6 ± 34.4	-206.0 ± 26.1	-238.0 ± 26.8	-311.2 ± 52.7	-295.6 ± 61.6	-216.7 ± 48.2
Desolvation energy	1.1 ± 1.6	3.2 ± 2.4	3.6 ± 3.4	-3.2 ± 2.4	3.9 ± 2.6	-4.9 ± 1.7	-2.2 ± 1.0	13.5 ± 3.3	5.3 ± 2.4	3.1 ± 8.3
Restraints violation energy	95.9 ± 19.4	80.6 ± 45.1	103.7 ± 17.9	60.1 ± 14.1	20.8 ± 14.1	119.5 ± 47.7	125.3 ± 37.8	63.4 ± 36.8	144.9 ± 43.9	89.6 ± 80.1
Buried Surface Area	1425.6 ± 47.1	1381.5 ± 34.2	1202.7 ± 96.3	1189.7 ± 75.7	1228.8 ± 35.2	1290.4 ± 113.7	1127.5 ± 7.2	1003.9 ± 44.7	915.4 ± 96.1	1073.0 ± 128.5
Z-Score	-1.9	-1.1	-0.5	-0.5	0	0.1	0.3	1.1	1.2	1.3
**(REN) Renin**	**Cluster 2**	**Cluster 3**	**Cluster 4**	**Cluster 10**	**Cluster 7**	**Cluster 5**	**Cluster 1**	**Cluster 6**	**Cluster 8**	**Cluster 9**
HADDOCK score	-67.3 ± 3.5	-66.8 ± 1.0	-64.3 ± 3.1	-63.9 ± 4.7	-62.2 ± 7.9	-61.7 ± 3.1	-52.5 ± 2.9	-49.0 ± 2.1	-35.6 ± 5.2	-34.4 ± 6.7
Cluster size	42	33	14	4	6	10	52	8	5	4
RMSD from the overall lowest-energy structure	2.2 ± 0.2	2.3 ± 0.0	2.1 ± 0.1	2.1 ± 0.1	2.0 ± 0.3	2.6 ± 0.0	3.4 ± 0.1	2.6 ± 0.1	3.2 ± 0.1	3.3 ± 0.1
Van der Waals energy	-46.1 ± 3.7	-33.8 ± 3.1	-34.2 ± 3.0	-40.8 ± 3.4	-44.0 ± 2.3	-34.3 ± 3.4	-26.9 ± 2.0	-24.7 ± 5.1	-17.9 ± 5.2	-16.1 ± 1.6
Electrostatic energy	-120.4 ± 22.7	-209.7 ± 17.6	-200.2 ± 15.7	-148.5 ± 10.9	-92.2 ± 47.6	-210.9 ± 12.9	-116.2 ± 16.7	-186.0 ± 6.0	-118.4 ± 9.9	-83.6 ± 34.2
Desolvation energy	-8.5 ± 3.4	6.6 ± 1.3	3.8 ± 1.1	3.1 ± 1.8	-13.3 ± 3.6	4.8 ± 0.8	-9.6 ± 0.6	1.5 ± 1.7	-2.7 ± 3.4	-9.1 ± 2.7
Restraints violation energy	114.0 ± 42.3	24.0 ± 16.9	61.2 ± 37.0	35.5 ± 8.1	135.3 ± 53.7	100.1 ± 24.4	71.6 ± 36.9	113.8 ± 26.7	86.6 ± 21.0	75.3 ± 39.6
Buried Surface Area	1203.1 ± 50.8	1226.3 ± 42.4	1084.7 ± 42.9	1269.3 ± 16.7	1235.6 ± 52.4	1282.4 ± 40.4	1035.7 ± 29.5	976.3 ± 49.6	921.7 ± 58.4	799.4 ± 77.9
Z-Score	-1	-0.9	-0.7	-0.7	-0.5	-0.5	0.3	0.6	1.7	1.8
